# Willingness to Receive the Booster COVID-19 Vaccine Dose in Poland

**DOI:** 10.3390/vaccines9111286

**Published:** 2021-11-05

**Authors:** Piotr Rzymski, Barbara Poniedziałek, Andrzej Fal

**Affiliations:** 1 Department of Environmental Medicine, Poznan University of Medical Sciences, 60-806 Poznań, Poland; bpon@ump.edu.pl; 2 Integrated Science Association (ISA), Universal Scientific Education and Research Network (USERN), 60-806 Poznań, Poland; 3 Collegium Medicum, Warsaw Faculty of Medicine, Cardinal Stefan Wyszyński University, 01-938 Warsaw, Poland; amfal@wp.pl

**Keywords:** COVID-19 vaccine, booster dose, vaccine hesitancy, mRNA vaccines, vector vaccines, SARS-CoV-2

## Abstract

COVID-19 vaccinations are essential to mitigate the pandemic and prevent severe SARS-CoV-2 infections. However, the serum antibody levels in vaccinated individuals gradually decrease over time, while SARS-CoV-2 is undergoing an evolution toward more transmissible variants, such as B.1.617.2, ultimately increasing the risk of breakthrough infections and further virus spread. This cross-sectional online study of adult Poles (*n* = 2427) was conducted in September 2021 (before a general recommendation to administer a booster COVID-19 vaccine dose in Poland was issued) to assess the attitude of individuals who completed the current vaccination regime toward a potential booster dose of the COVID-19 vaccine and identify potential factors that may influence it. Overall, 71% of participants declared willingness to receive a booster COVID-19 dose, with a low median level of fear of receiving it of 1.0 (measured by the 10-point Likert-type scale), which was increased particularly in those having a worse experience (in terms of severity of side effects and associated fear) with past COVID-19 vaccination. The lowest frequency of willingness to receive a booster dose (26.7%) was seen in the group previously vaccinated with Ad26.COV2.S. The majority of individuals vaccinated previously with mRNA vaccines wished to receive the same vaccine, while in the case of AZD1222, such accordance was observed only in 9.1%. The main reasons against accepting a booster COVID-19 dose included the side effects experienced after previous doses, the opinion that further vaccination is unnecessary, and safety uncertainties. Women, older individuals (≥50 years), subjects with obesity, chronic diseases, and pre-vaccination and post-vaccination SARS-CoV-2 infections, and those with a history of vaccination against influenza were significantly more frequently willing to receive a booster COVID-19 dose. Moreover, the majority of immunosuppressed individuals (88%) were willing to receive an additional dose. The results emphasize some hesitancy toward potential further COVID-19 vaccination in the studied group of Poles and indicate the main groups to be targeted with effective science communication regarding the booster doses.

## 1. Introduction

The COVID-19 pandemic has been met with unprecedented research responses that received significant economic and institutional support, enabling the development of numerous vaccine candidates, some of which underwent preclinical and clinical studies and were authorized at an unseen pace [1,2,3]. The outbreak of the first COVID-19 cases was reported in late December 2019 in China and an etiological factor, SARS-CoV-2, was molecularly characterized in January 2020, while the first vaccination campaigns were launched in December 2020 with 3.12 billion individuals (40.1% of the world population) receiving at least one dose of the COVID-19 vaccine till the beginning of September 2021. As of September 2021, four COVID-19 vaccines have received conditional marketing authorization in the European Union: mRNA vaccine BNT162b2 (BioNTech/Pfizer, Germany, Mainz/New York, NY, USA) given as two doses 21 days apart and mRNA-1273 (Moderna, Cambridge, MA, USA) given as two doses 28 days apart, as well as adenoviral vector vaccine AZD1222 (Oxford/AstraZeneca, UK/Sweden) given as two doses 4–12 weeks apart and Ad26.COV2.S (Janssen/Johnson & Johnson, Leiden, Netherlands/New Brunswick, NJ, USA), which is administered as a single dose [4,5,6,7].

The short-term results of clinical trials of different COVID-19 vaccines demonstrated their high efficacy against symptomatic SARS-CoV-2 infection [4,5,6], later confirmed by the first post-authorization, real-world observations [8]. However, accumulating evidence suggests that this efficacy gradually decreases due to two main factors: (i) a decline in serum anti-spike IgG antibody levels that occurs within a few months following the last vaccination [9,10], and (ii) the emergence of novel SARS-CoV-2 variants, classified as variants of interest (VOIs) and variants of concern (VOCs), such as B.1.617.2 (delta variant), that reveal higher transmissibility and can lead to higher viral loads in the upper respiratory airways [11,12]. The six-month follow-up of participants in the clinical trial of the BNT162b2 vaccine demonstrated that efficacy against infection decreased approx. 6% every two months [13]. However, these results should be cautiously extrapolated. With a cut-off date of 13 March 2021, the analysis could not assess the efficacy against the B.1.617.2 variant that became dominant globally since mid-June 2021. However, the real-world data clearly show the significantly decreased efficacy of various COVID-19 vaccines against this infection with this variant [14,15].

It should be stressed that although a significant decrease in protection against SARS-CoV-2 infection was noted, real-world data reassure the high level of vaccine efficacy against severe COVID-19 despite the emergence of highly transmissible variants with increased potency to induce breakthrough infections [15]. This likely highlights the role played by adaptive cellular immunity generated by vaccination. However, the potential of variants such as B.1.617.2 to induce similar viral loads in fully vaccinated and unvaccinated individuals within the first five days of infection [16,17,18] and the increased basic reproduction number for this variant (up to 8 compared to 4.0 for B.1.1.7 and <3.0 for variants dominant in 2020) [19,20] mean that it may, at some point, be inevitable to recommend a booster vaccine dose to control the spread of SARS-CoV-2 under pandemic conditions. In various countries, the decision to offer the additional dose to immunocompromised individuals has already been taken. It is well known that this group reveals weaker humoral and cellular responses to vaccines and more frequently is represented by non-responders. COVID-19 vaccines are no exception in this regard [21,22,23]. At the same time, studies, including randomized clinical trials, demonstrate that additional doses benefit individuals such as transplant recipients in terms of immune response and associate protection [24,25]. However, the odds for a diminished response to the COVID-19 vaccine also increase in the elderly, especially those ≥75 years old [26,27,28]. At the same time, they represent a high-risk group for severe COVID-19, indicating that a booster dose may be beneficial to provide them with additional protection from infection. Eventually, putting the spread of SARS-CoV-2 VOCs under better control may require booster doses for the general population, a strategy that Israel has already adopted—the first country in the world to do so. However, as highlighted by authorities such as the European Center for Disease Control and the European Medicines Agency, priority should still be given to vaccinating all eligible individuals who have not yet initiated or completed their recommended vaccination course [29]. The consensus report published in September 2021 in *The Lancet* argues against booster doses at this moment and highlights that although there is a decrease in vaccine efficacy against infection, unvaccinated individuals remain the main drivers of SARS-CoV-2 transmission [30]. Indeed, the priority should now be given to vaccinating unvaccinated individuals, especially in low-income countries, rather than optimizing the protection levels of wealthy populations [31]. Nevertheless, the discussions on booster COVID-19 vaccine doses will certainly continue within academia and outside. At the same time, the attitude toward this issue in different European populations remains unknown and urgently needs to be investigated.

This study aimed to assess the attitude of adult Poles who were fully vaccinated at the moment of study (September 2021) toward a potential booster COVID-19 vaccine dose, associated fear, and factors behind the unwillingness to receive it. The main research questions included: (i) What percentage of fully vaccinated individuals are willing to receive a booster dose? (ii) Which demographical groups are more and less willing to receive it? (iii) What are the main reasons against accepting a booster dose? (iv) How high is the level of fear of booster dose in the group willing to receive it? (v) Are there any preferences toward a specific COVID-19 vaccine to be given as a booster? Understanding the reasons for the rejection of potential booster doses is essential in shaping further science communication and building trust in COVID-19 vaccines.

## 2. Materials and Methods

### 2.1. Survey

This study was based on the anonymous, self-designed, and structured online questionnaire (Supplementary Materials) that was made available through a media release by the Polish Press Agency (the single largest source of news in Poland) and Wirtualna Polska (the most viewed online source of news in Poland), subsequently shared by a number of other media outlets and their associated social media profiles, leading to the snowball effect. Such online research is preferable to swiftly reaching a group of individuals during the pandemic [32] and has been successfully employed in previous investigations on vaccine hesitancy [33,34].

Specifically, the survey aimed to assess:

- The percentage of Poles fully vaccinated at the time of the study who would be willing to receive a booster dose of the COVID-19 vaccine (third dose in the case of mRNA vaccines and AZD1222, and second dose in the case of Ad26.COV2.S);
- The level of fear of booster COVID-19 vaccine dose (measured with a 10-point Likert-type scale, where 1—no fear, 10—very high level of fear) in individuals willing to receive it;
- The preferences toward specific COVID-19 vaccines (BNT162b2, mRNA-1273, AZD1222, or Ad26.COV2.S) to be administered as a booster dose and whether this corresponds to the COVID-19 vaccine given in the past;
- Primary reasons behind the unwillingness to receive a booster COVID-19 vaccine;
- The factors that are associated with willingness/unwillingness to receive a booster dose of the COVID-19 vaccine, including demographical characteristics (age, gender, body mass index, chronic diseases, presence of immunosuppression, SARS-CoV-2 infection status), as well as past experience with the COVID-19 vaccine evaluated as the severity level of side effects following each past dose and fear level accompanying these side effects (both measured with a 10-point Likert-type scale, where 1—no side effects or negligible side effects/no fear, 10—highly severe side effects/very high level of fear), and attitude toward influenza vaccination.

Recorded demographic data included age, gender, level of education, urban or rural residence, body mass index (BMI, calculated from collected data on weight and height), chronic diseases, immunosuppression, and status of SARS-CoV-2 infection. Since individuals vaccinating against influenza can reveal higher acceptance of the COVID-19 vaccine and a better understanding of repeated vaccination regimens [35,36], the attitude toward influenza vaccination (vaccinating annually, vaccinating infrequently, never vaccinating) was established for each participant.

The survey was available for one week between 8 and 15 September 2021, soon after the Polish Ministry of Health recommended an additional dose of mRNA vaccines exclusively for different groups of immunocompromised individuals, but before a recommendation on the use of a booster COVID-19 vaccine dose in in all adults was issued in Poland on 2 November 2021. The inclusion criteria for the study included: age ≥ 18 years old, Polish nationality, and status of the fully vaccinated individual at the time of the survey. During the study period, the number of COVID-19 doses administered in Poland amounted to 97 per 100 inhabitants. Overall, 19 million Poles (50% of the population) have been considered fully vaccinated after receiving two doses of mRNA vaccine (BNT162b or mRNA-1273) or adenoviral vector AZD1222 vaccine, or one dose of Ad26.COV2.S adenoviral vector vaccine (Janssen/Johnson & Johnson, Leiden, Netherlands/New Brunswick, NJ, USA).

Given the size of the target population (defined as a group of fully vaccinated individuals in Poland at the time of the study), it was calculated using Cochran’s formula [37] that at least 2401 eligible individuals should be surveyed to reach a margin level of 2% at the confidence level of 95%. Ten-point Likert-type scales to measure the level of vaccination fear and severity of side effects following the initial COVID-19 vaccination were chosen as they were successfully employed in various other cross-sectional investigations of vaccine hesitancy and attitudes [33,38,39,40,41]. The scales’ internal consistency reliability was determined with Cronbach’s alpha and demonstrated good reliability of α = 0.82–0.93.

### 2.2. Statistical Analysis

Statistica v.13.1 (StatSoft Inc., Tulsa, OK, USA) was used for data analysis. Non-parametric methods were applied because age and BMI did not meet the assumption of Gaussian distribution, while levels of fear and side effect severity were measured by the ordinal Likert-type scale. Differences between groups were assessed with the Mann–Whitney U test (two groups) or Kruskal–Wallis ANOVA (more than two groups). For nominal categorical variables, differences in frequencies were tested with Pearson’s χ2 test. The willingness to receive a booster dose of the COVID-19 vaccine and the level of associated fear were assessed in relation to age (<50/≥50 years old), gender, BMI, level of education (tertiary/other), place of living (urban/rural), immunosuppression (present/not present), chronic diseases (present/not present), history of SARS-CoV-2 infection (not infected/infected prior to vaccination/infected after at least one dose), past experience with COVID-19 vaccine (severity of side effects and level of accompanying fear), and history of vaccination against influenza. To account for alpha inflation and limit the probability of type 1 error, Bonferroni corrections were applied in all multiple comparisons. A *p*-value < 0.05 was considered statistically significant.

## 3. Results

### 3.1. Demographic Characteristics

The survey was completed by 2782 individuals, of which 2427 (87.3%) met the study criteria and were included in further analyses. The demographic characteristics of the studied group are summarized in Table 1. In general, most participants were <50 years old, had an increased BMI (54.9%, *n* = 1331), inhabited urban areas, had higher education, had no history of SARS-CoV-2 infection, and never received a vaccine against influenza. Approximately one-quarter of surveyed participants (*n* = 643) suffered from at least one chronic disease, with cardiovascular disease being most often declared. Immunosuppressed individuals constituted 6.2% (*n* = 150).

The majority of participants were vaccinated with BNT162b2 (60.3%, *n* = 1463), followed by AZD1222 (19.8%, *n* = 481), mRNA-1273 (12.2%, *n* = 296), and Ad26.COV2.S (7.7%, *n* = 187).

### 3.2. Willingness to Receive a Booster COVID-19 Vaccine Dose

Overall, 71.0% (*n* = 1724) of the surveyed participants declared a willingness to receive the potential booster dose of the COVID-19 vaccine, while 4.3% (*n* = 105) were unsure about it. The primary reason against it included the side effects experienced after previous doses (49.2%, *n* = 294), followed by the opinion that a booster dose is unnecessary (39.5%, *n* = 236) and safety uncertainties (22.4%, *n* = 134%). Willingness to receive the potential booster vaccine dose was declared most frequently by individuals previously vaccinated with AZD1222 (82.3%, *n* = 396), followed by BNT162b2 (72.7%, *n* = 1064) and mRNA-1273 (72.3, *n* = 214), while among those vaccinated with Ad26.COV2.S it was only 26.7% (*n* = 50) with 65.2% (*n* = 122) reporting no interest in further vaccination.

**Figure 1:**
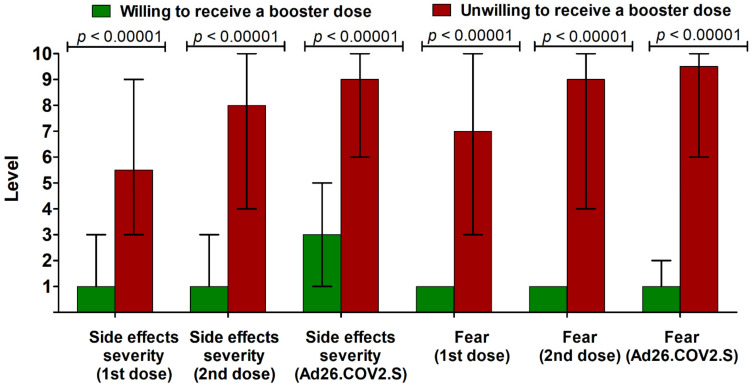
The median (interquartile range) level of side effect severity and associated fear (in 1–10 Likert-like scale) after previously received COVID-19 vaccines in individuals willing and unwilling to receive the potential booster dose (*n* = 2322). Subjects who were unsure about receiving a booster dose were excluded from this analysis (*n* = 105).

The willingness to receive the booster dose of the COVID-19 vaccine was significantly higher in older subjects (≥50 years old), women, individuals with obesity and chronic diseases, and those with a history of influenza vaccination. Moreover, the majority of immunosuppressed individuals (88%) declared an interest in an additional COVID-19 vaccine dose. Subjects with a history of SARS-CoV-2 infection prior to vaccination were less frequently interested in the booster dose than those without a COVID-19 history, while individuals infected after receiving at least one dose of the COVID-19 vaccine were mostly against it (Table 2). The place of living and level of education were not associated with analyzed willingness. Individuals unwilling to receive a booster dose reported higher severity of side effects following the previous COVID-19 vaccine doses and experienced a higher level of fear associated with these side effects (Figure 1).

### 3.3. Fear of a Booster COVID-19 Vaccine Dose

**Figure 2:**
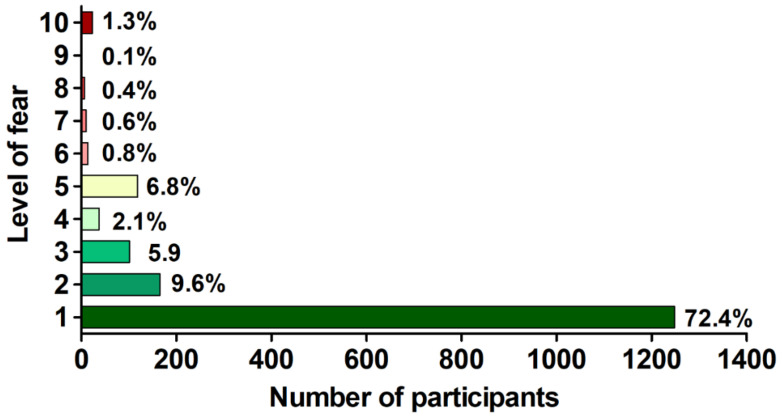
The distribution of level of fear (in 1–10 Likert-like scale) associated with a booster COVID-19 vaccine dose in participants willing to receive it (*n* = 1724).

The general median (interquartile range, IQR) level of fear of the booster COVID-19 vaccine in those willing to receive it (defined by the 10-point Likert-type scale) was 1.0 (1.0–2.0), with only 3.2% (*n* = 55) of individuals declaring a level >5 (Figure 2).

**Figure 3:**
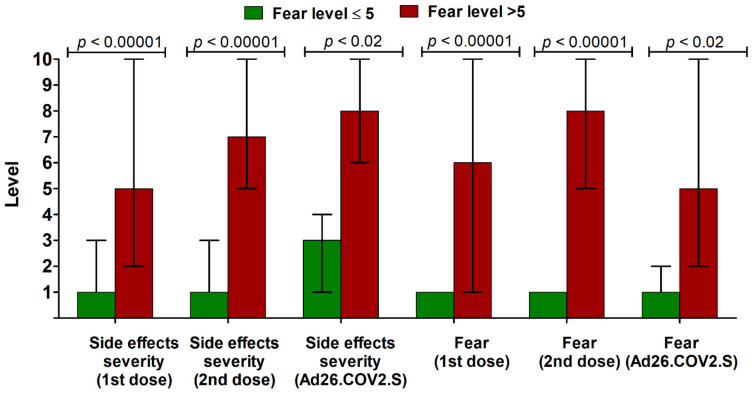
The median (interquartile range) level of side effect severity and associated fear (in 1–10 Likert-like scale) after previously received COVID-19 vaccines in individuals declaring lower (<5) and increased (≥5) level of fear of the potential booster vaccine dose (*n* = 1724).

Moreover, the fear of the potential booster COVID-19 vaccine dose did not differ between participants previously vaccinated with BNT162b2, mRNA-1273, AZD1222, and Ad26.COV2.S (*p* > 0.05; Kruskal–Wallis ANOVA). It was, however, more frequently increased in younger individuals (<50 years old) and in subjects infected with SARS-CoV-2 after receiving at least one dose of the COVID-19 vaccine. Other demographical parameters were not associated with a higher level of fear (Table 3). Individuals displaying fear > 5 were characterized by a worse past experience with COVID-19 vaccines in terms of the level of side effect severity and associated level of fear (Figure 3).

### 3.4. Preferences of Type of Booster COVID-19 Vaccine Dose

**Figure 4:**
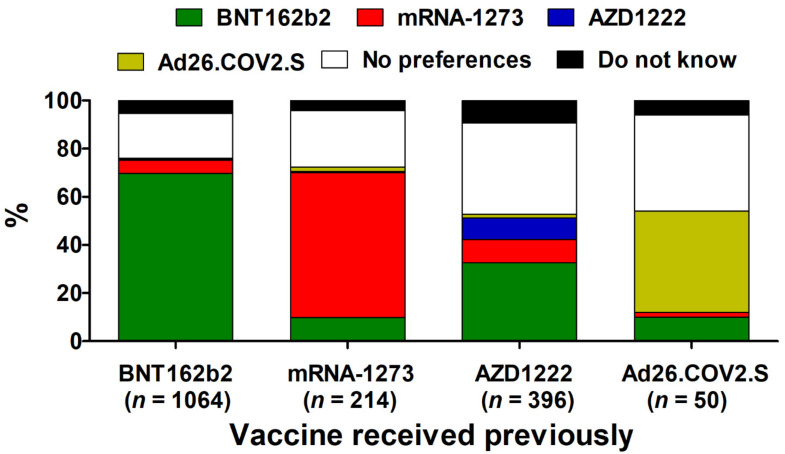
The preference of the specific COVID-19 vaccine to be used as the potential booster dose in the groups previously vaccinated with BNT162b, mRNA-1273, AZD1222, and Ad26.COV2.S (*n* = 1724).

Participants who declared their willingness to receive a booster COVID-19 vaccine dose did not always prefer immunization with the same vaccine as administrated previously. Figure 4 shows the preferences of surveyed individuals toward a particular COVID-19 vaccine they wish to receive as the potential booster dose. In general, 24.2% (*n* = 418) declared no specific preferences in this regard, while 6.1% (*n* = 106) could not decide at the moment of the survey. However, most participants who completed their initial regime with BNT162b2 and mRNA-1273 wished to receive the potential booster dose with the same vaccine (69.8%, *n* = 743 and 60.3%, *n* = 129, respectively). In the case of Ad26.COV2.S, 42.0% (*n* = 47) of surveyed individuals were interested in receiving it as a booster dose, while in the case of AZD1222, accordance was observed only in 9.1% (*n* = 36) of subjects.

Compared to individuals preferring to receive the same vaccine, those choosing a different vaccine reported a higher level of side effect severity after the first vaccine dose (median (IQR) 1 (1–4) vs. 2 (1–5), *p* = 0.000028; Mann–Whitney U test), a higher level of associated fear (1 (1–2) vs. 1 (1–4), ), *p* = 0.00028; Mann–Whitney U test), and a higher level of severity of side effects following a single Ad26.COV2.S dose (2 (1–4) vs. 8 (5–10), *p* < 0.00001; Mann–Whitney U test) and associated fear (1 (1–1) vs. 8 (5–10, *p* < 0.00001; Mann–Whitney U test).

## 4. Discussion

Convincing Polish citizens to receive a COVID-19 vaccine has already been a challenging task. In November 2020, only 20% of Poles declared a willingness to vaccinate, and this figure increased to 55% in mid-February 2021 [42,43]. However, during the first four months of 2021, approximately 30% declared a lack of willingness to receive any COVID-19 vaccine, with the main concern related to the potential side effects [44]. Until the present study was initiated, only 50% of the Polish population was fully vaccinated (and 51% received at least one dose) despite extensive science communication efforts and the worsening pandemic situation in the country, encompassing nearly 1.6 million new cases and 47 thousand deaths between January and September 2021. The present study highlights that public acceptance of the booster dose of the COVID-19 vaccine may also be met with some obstacles as approx. 30% of surveyed individuals declared no willingness to receive it. On the other hand, during the survey period, no recommendation to administer booster doses of vaccines to fully vaccinated individuals in the general population was issued in Poland. Yet, most of the participants already expressed a willingness to receive it when possible, with generally a very low level of associated fear. In this context, the findings of the present study are encouraging, particularly given anti-vaccine trends observed prior to the COVID-19 pandemic, reflected by the growing percentage of parents who refuse immunization for their children or by the very low (<5%) influenza vaccination coverage rate in the general population [45,46].

Importantly, the present study shows that the majority of individuals representing groups of high COVID-19 risk, including elderly, obese subjects and those suffering from chronic diseases such as cancer, cardiovascular diseases, or chronic kidney diseases, are prepared for the potential further vaccine dose and will likely follow the updated guidelines on COVID-19 vaccination strategies. It is also reassuring to confirm that the majority of immunosuppressed patients, who represent a priority group for a boosting strategy [31], are willing to receive the additional vaccine dose as it should improve their overall situation during the COVID-19 pandemic.

Initial studies have shown that women may display greater vaccine hesitancy and a higher level of fear related to COVID-19 vaccination [33,47]. The potential explanation behind these observations could be linked to psychological gender differences [48,49] and the more cautious approach of women to acceptance of innovative medical technologies [50,51]. However, the present study demonstrated not only that women do not show a higher level of fear of the potential booster COVID-19 dose, but contrarily, they are more willing to receive it. This indicates a potential shift in women’s perception of COVID-19 vaccines as an established preventive measure. In turn, previous studies have clearly shown that women demonstrate better compliance with public health policies and non-pharmacological preventive measures during the COVID-19 pandemic as they are more likely to define it as a serious health problem [52].

Interestingly, the present study also found a higher willingness to receive the booster dose of the COVID-19 vaccine in those who vaccinate against influenza. This highlights that these individuals reveal a better understanding of the need for repeated vaccination and most likely will also accept it in the case of COVID-19 vaccines. Whether this is also the case in countries with much higher influenza vaccination coverage levels (e.g., the United Kingdom) requires additional studies.

There was also a lower willingness to receive the potential booster COVID-19 vaccine dose among individuals infected with SARS-CoV-2 after the administration of at least one dose. This may partially be due to a decreased trust in vaccines that were reported to have a very high efficacy at preventing symptomatic infection in short-term clinical trial observations [4,5,6,7]. It is essential to emphasize that even though vaccines’ effectiveness in preventing infections can decrease over time [13], they still confer a high level of protection against severe COVID-19, hospitalization, and death [15]. On the other hand, individuals infected post-vaccination may not necessarily need any booster dose. It is known that the vaccination of convalescent patients improves the cellular and humoral response, also regarding the neutralization of VOCs [53,54,55]. Whether this is also the case in individuals infected post-vaccination remains, however, to be elucidated.

The present study demonstrated that the participants’ preferences toward a specific COVID-19 vaccine to be used as a booster dose do not necessarily match the vaccine used previously. Preferences to use a different vaccine were usually associated with relevant side effect severity after the first (or second) dose, highlighting the role played by past experiences in vaccine perception and trust. The highest level of accordance was seen for both mRNA vaccines. This may be, at least partially, due to the fact that the general administration of the third dose of one of the mRNA vaccines, BNT162b2, was already initiated in Israel when the present study was conducted. In contrast, at the time of the present study, no recommendation for the booster dose of vector COVID-19 vaccines was issued anywhere in the world. Moreover, previous studies have shown that mRNA vaccines have the highest level of acceptance in the Polish population [33], and the present findings confirm this.

On the other hand, over 40% of those previously vaccinated with AZD1222 wished to receive the potential booster dose of a different vaccine, with the majority choosing BNT162b2. The low level of accordance found for AZD1222 is likely due to safety concerns. This vaccine has been linked with rare thrombotic events associated with thrombocytopenia that received high media coverage in Europe and led several countries to suspend its use [56]. As shown previously, the level of trust in AZD1222 was decidedly lower in the Polish population when compared to mRNA vaccines [33]. Moreover, AZD1222 was also receiving generally bad press in Europe during the first months of 2021 due to reduced deliveries by the manufacturer and was subject to systemic misinformation spread via online social media [57]. Interestingly, the majority of the individuals vaccinated with Ad26.COV2.S did not express willingness to receive a booster dose of any available vaccine. In a clinical trial, the second dose of Ad26.COV2.S has been found to markedly increase humoral responses that may translate into sustained protective efficacy [58]. However, this vaccine was recommended for use as a single dose and could be perceived as a convenient alternative compared to other COVID-19 vaccines, which require two doses given apart. The US survey showed that single-dose vaccination is more preferred than a two-dose regimen [59]. Therefore, a potential future necessity to receive another dose of Ad26.COV2.S may not be initially well understood and may require some communication efforts.

Study limitations should be stressed. This was an online survey excluding the verification of selected data on more objective grounds. The levels of side effect severity and associated fear were measured using a 10-point Likert-type scale, which is prone to subjectiveness. Although there was a similar share of women and men (which is often not the case in similar cross-sectional surveys) and elderly subjects were relatively well represented in the present study, some subsets were under-represented, e.g., individuals with other than tertiary education or inhabitants of rural areas. In turn, online research may attract the attention and willingness of more young, better-educated subjects inhabiting urban areas, as also reflected by the demographical structure of the group studied in the present investigation. Moreover, a volunteer bias cannot be fully excluded —an anonymous online survey may attract attention of those with definite judgments on a booster dose more than individuals who are hesitant or have no opinion. Last but not least, not all declarations given in the study by participants may be reflected in actual future decisions as they can be affected by various factors, including local dynamics of the COVID-19 pandemic, close experiences with COVID-19, as well as the quality of media content on vaccines.

## 5. Conclusions

The present cross-sectional online study assessed the willingness and attitudes toward a potential booster dose of the COVID-19 vaccine in a group of Poles. The results indicate that a relatively significant share of those who previously decided to receive a COVID-19 vaccine could be against it. The main reasons against accepting a booster COVID-19 dose included the side effects experienced after previous doses, the opinion that further vaccination is unnecessary, and safety uncertainties. Identifying specific groups with the lowest level of acceptance of further COVID-19 vaccination is essential for effective science communication and building general trust in vaccines, especially if one considers that booster COVID-19 vaccine doses may eventually be inevitable to put the spread of SARS-CoV-2 under better control.

## Figures and Tables

**Table 1:** Summary of the demographic characteristics of the studied group (*n* = 2427).

Parameter	Statistics
**Age** (years), median, interquartile Range (min–max)	44.0, 36.0–58.0 (18–99)
Aged <50, % (n)	62.3 (1513)
Aged ≥50, % (n)	37.7 (914)
**Gender**	
Female, % (n)	50.7 (1231)
Male, % (n)	49.3 (1196)
**BMI** (kg/m^2^), median, interquartile range (min–max)	25.5, 22.7–28.9 (14.0–56.1)
Underweight (<18.5), % (n)	2.8 (69)
Normal weight (18.5–24.9), % (n)	42.3 (1027)
Overweight (25.0–29.9), % (n)	34.8 (844)
Obesity (≥30.0), % (n)	20.1 (487)
**Place of living**	
Urban, % (n)	87.0 (2112)
Rural, % (n)	13.0 (315)
**Education**	
Primary, % (n)	1.5 (37)
Secondary, % (n)	3.5 (85)
Vocational, % (n)	24.4 (591)
Tertiary, % (n)	70.6 (1714)
**Immunosuppression, % (n)**	6.2 (150)
**Chronic disease, % (n)**	26.5 (643)
Diabetes, % (n)	7.8 (190)
Cancer, % (n)	2.9 (70)
Cardiovascular disease, % (n)	12.2 (296)
Chronic kidney disease, % (n)	2.1 (52)
Chronic pulmonary disease, % (n)	3.4 (83)
Asthma, % (n)	7.0 (170)
**SARS-CoV-2 infection status**	
Infected prior to vaccination, % (n)	13.4 (324)
Infected between 1st and 2nd dose, % (n)	1.5 (37)
Infected after full vaccination, % (n)	6.1 (149)
No history of infection, % (n)	79.0 (1917)
**Influenza vaccine status**	
Vaccinated annually, % (n)	18.4 (447)
Vaccinated irregularly, % (n)	25.2 (611)
Never vaccinated, % (n)	56.4 (1369)

**Table 2:** The frequency of willingness and unwillingness to receive the potential booster COVID-19 vaccine dose in different demographical groups in the studied population (*n* = 2322). Subjects who were unsure about receiving the booster dose were excluded from this analysis (*n* = 105).

Parameter		Willing to Receive (*n* = 1724)	Unwilling to Receive (*n* = 598)	*χ*^2^ Test *p*-Value (with Bonferroni Correction)
		%		
**Age**	<50	68.1	31.9	<0.00001
	≥50	84.3	15.7	
**Gender**	Female	80.0	20.0	<0.00001
	Male	68.4	31.6	
**BMI**	Underweight	63.1	36.9	<0.00001
	Normal BMI	70.1	29.9	
	Overweight	74.0	26.0	
	Obesity	84.7	15.3	
**Place of living**	Urban	74.8	25.2	>0.05
	Rural	70.4	29.6	
**Education**	Tertiary	74.4	25.6	>0.05
	Other	73.9	26.1	
**Immunosuppression**	Yes	88.0	12.0	0.0012
	No	73.3	26.7	
**Chronic disease**	Yes	86.5	13.5	<0.00001
	No	69.9	30.1	
**SARS-CoV-2** **infection status**	Not infected	80.5	19.5	<0.00001
	Infected prior to vaccination	66.9	33.1	
	Infected after at least 1 dose	21.5	78.5	
**Influenza** **vaccine status**	Vaccinated annually	92.4	7.6	<0.00001
	Vaccinated irregularly	86.5	13.5	
	Never vaccinated	62.5	37.5	

**Table 3:** The percentage of individuals in the studied group (*n* = 1724) with the level of fear of >5 (in 1–10 Likert-like scale) of the potential booster COVID-19 vaccine in relation to different demographical parameters.

**Parameter**		Fear ≤5 (*n* = 1669)	Fear >5 (*n* = 55)	*χ*^2^ Test *p*-Value (with Bonferroni Correction)
		%		
**Age**	<50	95.7	4.3	0.033
	≥50	98.3	1.7	
**Gender**	Female	97.2	2.8	>0.05
	Male	96.3	3.7	
**BMI**	Underweight	95.1	4.9	>0.05
	Normal BMI	97.1	2.9	
	Overweight	96.3	3.7	
	Obesity	97.2	2.8	
**Place of living**	Urban	96.7	3.3	>0.05
	Rural	96.8	3.2	
**Education**	Tertiary	97.5	2.5	>0.05
	Other	95.0	5.0	
**Immunosuppression**	Yes	92.8	7.2	>0.05
	No	97.2	2.9	
**Chronic disease**	Yes	97.6	95.1	>0.05
	No	2.4	4.9	
**SARS-CoV-2** **infection status**	Not infected	97.8	2.2	<0.00001
	Infected prior to vaccination	95.5	4.5	
	Infected after at least 1 dose	63.2	36.8	
**Influenza** **vaccine status**	Vaccinated annually	96.8	3.2	>0.05
	Vaccinated irregularly	98.6	1.4	
	Never vaccinated	95.7	4.3	

## Data Availability

The data presented in this study are available from the corresponding author on reasonable request.

## Supplementary Materials

The following are available online at https://www.mdpi.com/article/10.3390/vaccines9111286/s1: Translated Questionnaire.
