# Supplementary material for: Willingness to Receive the Booster COVID-19 Vaccine Dose in Poland
Source: Vaccines (Basel). 2021 Nov 5;9(11):1286. doi: 10.3390/vaccines9111286 (PMC8624071; doi:10.3390/vaccines9111286)
Supplement: Supplementary file 1 [file vaccines-09-01286-s001.zip › vaccines-1407847-supplementary.pdf]

TRANSLATED QUESTIONNAIRE

1. **Have you been fully vaccinated against COVID-19 (received two doses of BioNTech-Pfizer, Moderna or AstraZeneca COVID-19 vaccine or a single dose of Janssen/Johnson&Johnson)?**
   Yes No

2. **Which COVID-19 vaccine have you been vaccinated with?**
   BioNTech-Pfizer (2 doses) Moderna (2 doses)
   AstraZeneca (2 doses) Janssen/Johnson&Johnson (1 dose)

3. **Please evaluate the severity of side effects that occurred after receiving your COVID-19 vaccine**
   (1 - no side effects/negligible side effects, 5 - medium severity, 10 - very high severity)

	1	2	3	4	5	6	7	8	9	10
First dose										
Second dose										

(If the answer " Janssen/Johnson&Johnson (1 dose)" in question #2

	1	2	3	4	5	6	7	8	9	10
Single dose (Janssen/Johnson&Johnson)										

4. **Please evaluate the level of fear accompanying the side effects that occurred after receiving your COVID-19 vaccine**
   (1 - no fear/very low level of fear, 5 - medium fear, 10 - very high fear)

	1	2	3	4	5	6	7	8	9	10
First dose										
Second dose										

(If the answer " Janssen/Johnson&Johnson (1 dose)" in question #2

	1	2	3	4	5	6	7	8	9	10
Single dose (Janssen/Johnson&Johnson)										

5. **Are you willing to receive the potential additional dose of the COVID-19 vaccine if it would be made available (third dose in the case of vaccinated with BioNTech/Pfizer, Moderna or AstraZeneca vaccines and second dose in the case of vaccinated with Janssen/Johnson&Johnson vaccine)?**
   Yes No Don't know

6. **(If the answer "yes" in question #5) Which vaccine would you like to receive as the additional COVID-19 vaccine dose?**
   It doesn't matter I don't know BioNTech/Pfizer
   Moderna AstraZeneca Janssen/Johnson&Johnson

7. **(If the answer "yes" in question #5) Please evaluate the level of fear associated with receiving the potential additional dose of the COVID-19 vaccine?**
   (1 - no fear/very low level of fear, 5 - medium fear, 10 - very high fear)

	1	2	3	4	5	6	7	8	9	10
Level of fear										

8. **(If the answer "no" in question #5) Why are you not willing to receive the additional dose of the COVID-19 vaccine dose?**
   I don't think it is necessary Due to safety concerns
   Due to side effects after previous doses of COVID-19 vaccine

9. **Have you been infected with the SARS-CoV-2?**
   No
   Yes, prior to vaccination
   Yes, between 1st and 2nd dose of vaccine (for those vaccinated with BioNTech/Pfizer, Moderna or AstraZeneca vaccines)
   Yes, after receiving all required doses (two doses of BioNTech/Pfizer, Moderna or AstraZeneca vaccines or a single dose of Janssen/Johnson&Johnson vaccine)

10. **Do you vaccinate against influenza?**
    Yes, regularly (annually)
    Yes, irregularly
    No, I was never vaccinated against the influenza

11. **Do you suffer from immune deficiency (primary or secondary)?**
    Yes No

12. **Do you suffer from any of these diseases?**
    Diabetes Cancer Cardiovascular disease Chronic pulmonary disease
    Chronic kidney disease Asthma None of these

13. **Your age** (in years):......

14. **Your gender:**
    Female Male

15. **Your weight** (in kilogram):......

16. **Your height** (in centimeters):......

17. **Your education level**
    Primary Secondary Vocational Tertiary

18. **Your place of living**
    Urban Rural
